# Identifying modifiable factors associated with COVID-19 vaccine hesitancy and acceptance among cancer patients in Jordan

**DOI:** 10.3389/fonc.2023.1281994

**Published:** 2023-11-28

**Authors:** Rama AlMasri, Mahmoud Al Masri, Rula Darwish, Khawla Ammar, Yasmin Safi

**Affiliations:** ^1^ Department of Surgery, King Hussein Cancer Center, Amman, Jordan; ^2^ School of Medicine, The University of Jordan, Amman, Jordan; ^3^ School of Pharmacy, The University of Jordan, Amman, Jordan; ^4^ Office of Research and Scientific Affairs, King Hussein Cancer Center, Amman, Jordan

**Keywords:** COVID-19 vaccine, coronavirus, vaccine acceptance, vaccine hesitancy, public health, vaccination, cancer patients, modifiable factors

## Abstract

**Introduction:**

Vaccines stand amongst the most effective medical interventions for the management of infectious diseases, and are pivotal tools for public health. The acceptance of vaccines is heavily influenced by perceptions of efficacy, safety and other modifiable factors.

**Purpose:**

This cross-sectional study sought to identify and examine the modifiable factors that can help address COVID-19 vaccine hesitancy and acceptance among cancer patients.

**Methods:**

The study was conducted between February and April 2021 using an online survey questionnaire comprising of four domains. The survey was administered to cancer patients in Jordan.

**Results:**

Among the 1,029 cancer patients who completed the online questionnaire (response rate= 73%), 58% (n=597) expressed willingness (intent) to take the vaccine. Notably, 72.5% (n=433) of those intending to take the vaccine were currently undergoing treatment. Knowledge and awareness played a significant role, with 54.3% considering them essential for vaccine acceptance. Fear of infection significantly influenced vaccine acceptance (p<0.001), with 66.8% expressing concern about potential infections. Peer encouragement was also a crucial factor, as 82.4% regarded it as an important driver for influencing vaccine acceptance (p<0.001).

**Conclusion:**

Peer encouragement, awareness, and fear emerged as the primary modifiable factors associated with greater vaccine acceptance by patients with active malignancies. Study results suggest that providing personalized and tailored information about vaccinations, focusing on safety and potential interactions with cancer and its treatment, are potentially excellent strategies for improving vaccine acceptance among cancer patients.

## 1 Introduction

Vaccine is among the most effective medical interventions for preventing and managing infectious diseases (1). The emergence of Coronavirus disease 2019 (COVID-19) has caused over 758,000,000 confirmed cases and 6,859,093 recorded deaths as of 2023, as reported by the World Health Organization (WHO) (2, 3). In Jordan, there are over 14,000 COVID-19 deaths as of 2023 (2). Some populations, such as cancer patients, are known to be particularly vulnerable to COVID-19 infection. Individuals with cancer have been shown to experience worse clinical outcomes and increased mortality from COVID-19, particularly those who are receiving active therapy or have advanced malignancy (4, 5). Although, there is substantial evidence in support of COVID-19 vaccines’ effectiveness, especially at preventing infection and severe disease (6–8), many people still express hesitancy towards using them (9, 10).

Hesitancy affects a wide range of people, ranging from those who absolutely reject all vaccinations to those doubt vaccines in special circumstances (2, 10). Vaccine hesitancy is complex and context-specific, varying across time, place and vaccine type. It is influenced by factors such as complacency, convenience and confidence. Most of these factors that influence vaccine hesitancy can be categorized either as modifiable or non-modifiable (11). The former group includes media impact, social acceptance, and worries about safety and efficacy, while the latter group includes disease and patient characteristics. Due to its unfamiliarity, lack of evidence about its efficacy at the time of introduction, and uncertainty about the long-term safety profile, cancer patients are often more prone to scrutinize but also accept the COVID-19 vaccine. Evidence shows that patients with active malignancies are more likely to hold misconceptions about contraindications to receiving the COVID vaccine due to their malignant disease (11). Fear of developing COVID-19 symptoms and infection have been known to help boost vaccine acceptance or adoption among cancer patients (12). The purpose of this study was to assess the willingness (intent) of cancer patients to receive the COVID-19 vaccine and describe possible strategies to improve vaccine acceptance based on their perceptions.

## 2 Materials and methods

The study utilizes a cross-sectional observational design to assess the attitude of cancer patients towards the COVID-19 vaccine, and pinpoint possible factors leading to vaccine hesitancy and poor acceptance of this medical intervention. Study participants were: (a) randomly selected from a pool of patients with active malignancies at the King Hussein Cancer Center, (b) over the age 18 years, and (c) were able to provide informed consent, during the 2-month sample period from February until April 2021. The study took place shortly after Jordan’s Food and Drug Administration (JFDA) approved the use of a number of vaccines for preventing the spread of COVID-19 in February 2021. Consent to participate in the study was obtained verbally from all participants who enrolled in the study. They were contacted by phone and were informed that study participation was voluntary and they can withdraw at any stage of the study. A link to the survey was sent to all participants after consenting. The online questionnaire was self-administered; follow ups were conducted within 3 days if no response had been received.

### 2.1 Survey questionnaire

A self-administered questionnaire was created to assess the perceptions of cancer patients towards the COVID-19 vaccine shortly after its arrival in Jordan. A literature review and a discussion was conducted with a group of experts to develop a questionnaire that contained question items appropriate for the target group: cancer patients. Face and content validity were tested with specialists involved with cancer patients during the COVID-19 pandemic including: physicians, nurses, psychosocial workers, survey specialists, clinic coordinators, statisticians and patients. The final version of questionnaire comprised 4 domains: demographics and disease characteristics, history of COVID-19 infection, vaccine awareness, and vaccine hesitancy. A majority of the questions required short answers or dichotomous (yes/no) responses, allowing for a more complete dataset (i.e., responses without excess missing data).

The first Domain consisted of questions about patient demographics and disease characteristics stratified by survey participant groups. Collected demographic information included age, gender, marital status, number of children (if applicable), monthly income, level of education, and occupation. Disease characteristics included confirmed diagnosis, treatment modalities, and current tumor stage. The second domain of questions asked about COVID-19 infection, including previous infection and associated symptoms. One question asked about patient’s fear of getting the coronavirus infection. In the third domain, 5 question items were used to assess patient awareness of vaccines and their baseline vaccine practices (e.g., previous seasonal flu vaccination practice, knowledge of the COVID-19 vaccine itself). The fourth and final domain of questions asked about possible vaccine hesitancy, their experience with the vaccination process, and the motives behind their reluctance.

### 2.2 Statistical tools

Study data were analyzed using IBM SPSS statistical software, version 28.0. The descriptive analysis reported on sample characteristics by frequency and percentage. The sample was divided into two groups based on their willingness (intent) to receive the COVID-19 vaccine: those willing (did intend to receive) and those not willing (did not intend to receive). A comparative analysis was conducted between these groups, utilizing cross tabulations for categorical data and employing Chi-Square or Fisher exact tests to assess for associations. Univariate tests were carried out to identify variables (confounding factors) that were included in the binary logistic multivariable regression analysis, this model was used to identify and describe statistically significant predictors of COVID-19 vaccine acceptance. The dependent variable for the model was operationalized as a binary response (Yes & No).A p value of < 0.05 was considered statistically significant.

## 3 Results

Among the 1410 participants who received the survey link, 1029 patients completed the survey for a response rate of 73%. **Table 1** shows the sample demographics: 495 males (48%), 534 females (52%), 74 (7%) who were single, 775 (75%) married, 67 divorced (6%), and 113 (10%) widowed. For the sample’s income distribution, 48% had a monthly income of less than 500 JOD (Jordanian Dinar), while 22% had a monthly income ranging between 500 and 1000 JOD. Those with a monthly income exceeding 1000 JOD constituted 20% of the total sample. Educational attainment was distributed as follows, 52% held an undergraduate degree, 16.9% had a postgraduate degree, 10% had primary education, 20% had tertiary education and 1% had secondary education. For work status, 49.9% were full-time employees, 13.3% worked part-time, 26.8% were housewives, 7.5% were retired, and 2.3% were unemployed.

**Table 1:** Sociodemographic characteristics of survey participants, from a pool of cancer patients at the King Hussein Cancer Center, Jordan (Feb-April 2021).

Characteristic		N=1029
Gender	Male	495 (48%)
	Female	534 (52%)
	Total	1029
Marital Status	Single	74 (7%)
	Married	775 (75%)
	Divorced	67 (6%)
	Widow	113 (10%)
	Total	1029
Income	Less than 500 JD	492 (48%)
	500- 1000 JD	326 (22%)
	More than 1000 JD	211 (20%)
	Total	1029
Education	Primary Education	96 (10%)
	Secondary Education	13 (1%)
	Tertiary education	209 (20%)
	Undergraduate degree	537 (52%)
	Postgraduate degree	174 (16.9%)
	Total	1029
Work	Part-time	137 (13.3%)
	Full-time	514 (49.9%)
	Housewife	276 (26.8%)
	Unemployed	24 (2.3%)
	Retired	78 (7.5%)
	Total	1029
	Surgery	503 (48.8%)
Previous treatment	Chemotherapy	366 (35.5%)
	Hormonal therapy	78 (7.5%)
	Radiotherapy	5 (<1%)
	Targeted therapy	2 (<1%)
	None	75 (7.2%)
	Total	1029
	Surgery	294 (28.5%)
Current treatment	Chemotherapy	252 (24.4%)
	Radiotherapy	162 (15.7%)
	Targeted therapy	55 (5.3%)
	Hormonal therapy	35 (3.4%)
	BMT	8 (<1%)
	None	223 (21.6%)
	Total	1029
Stage	I	229 (22.2%)
	II	313 (30.4%)
	III	397 (38.5%)
	IV	59 (5.7%)
	Total	998
Treatment stage	Pre-treatment	67 (6.5%)
	Post treatment	145 (14%)
	Active treatment	817 (79.3%)
	Total	1029

When asked about previous treatments, 48.8% had undergone surgery, 35.5% had received chemotherapy, 7.5% had received hormonal therapy, a small percentage had received radiotherapy (less than 1%), or targeted therapy (less than 1%), and 7.2% reported no previous treatment. For current treatment status, 21.6% reported no current treatment, 28.5% had undergone surgery recently, 24.4% received chemotherapy, and a small percentage received radiotherapy (15.7%), hormonal therapy (3.4%), targeted therapy (5.3%), or bone marrow transplantation (BMT, less than 1%).

Cancer stages among the 998 survey participants (there were some missing data in the overall sample) were as follows: 22.2% were in stage I, 30.4% in stage II, 38.5% in stage III, and 5.7% in stage IV. Additionally, 6.5% were in the pre-treatment stage, 14% were in the post-treatment stage, and 79.3% were in the active treatment stage.

**Figure 1:**
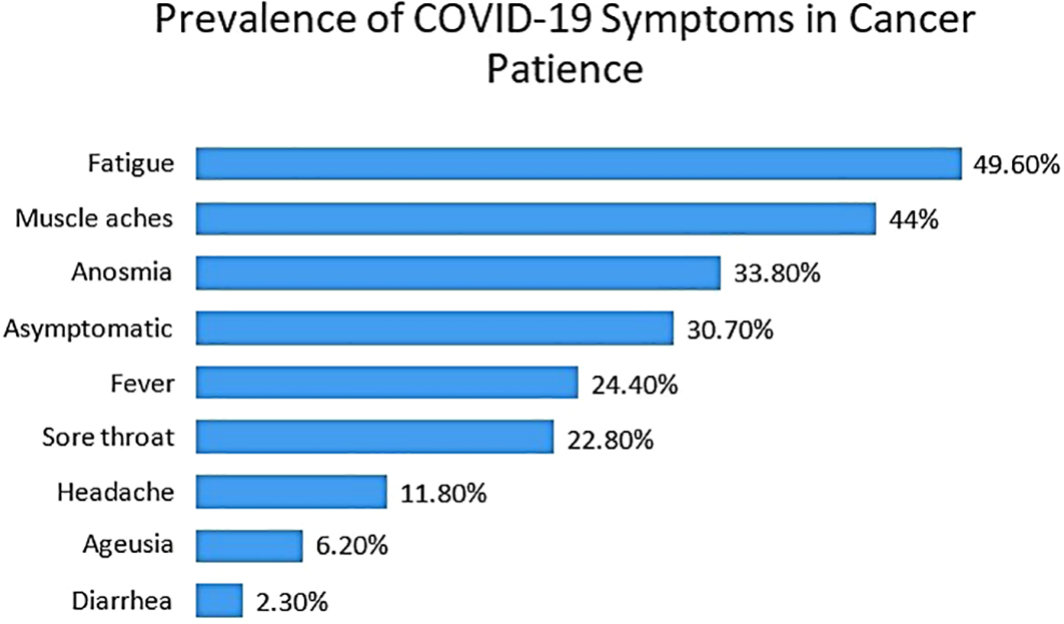
Prevalence of COVID-19 symptoms among patients at the King Hussein Cancer Center, Jordan (Feb-April 2021).

Of the total sample, 127 patients (12.3%) were diagnosed with COVID-19 infection. The most frequently reported symptom was fatigue, affecting 49.6% of them, followed by muscle ache (44%), anosmia (33.8%), fever (24.4%), sore throat (22.8%), and headache (11.8%). Less common symptoms were diarrhea, reported in 6.2%, and Ageusia, reported in 2.3%. Notably, 37.7% of the patients either experienced very mild symptoms that went unrecognized or were entirely asymptomatic. These results highlight the wide range of presenting symptoms associated with COVID-19 among cancer patients in Jordan. (**Figure 1**).

Among survey participants, 432 (42%) were not willing (did not intend) to take the vaccine, while 597 (58%) expressed a willingness (intent) to take the vaccine. Comparison between these two groups revealed no significant differences by demographic characteristics such as age, gender distribution, number of children, marital status, income, type of work, and previous treatments status (**Table 2**). In addition, health practices such as receiving the seasonal flu vaccine were found to be similar between the two groups.

**Table 2:** Comparison of patients who were willing versus those who were not willing to take the COVID-19 vaccine, King Hussein Cancer Center, Jordan (Feb-April 2021).

Variables		Will not take COVID 19 Vaccine N (%) 432 (42%)	Will take COVID 19 vaccine N (%) 597 (58%)	*P*
Age	Mean (SD)	50.51(14.17)	51.55(13.3)	0.227
	Min - Max	18 - 86	18 - 86	
Gender	Male	217 (43.8%)	278 (56.2%)	0.245
	Female	215 (40.3%)	319 (59.7%)	
Marital Status	Single	35 (47.3%)	39 (52.7%)	0.433
	Married	319 (41.2%)	456 (58.8%)	
	Divorced	33 (49.3%)	34 (50.7%)	
	Widow	45 (39.8%)	68 (60.2%)	
Income	< 500 JD	202 (41.1%)	290 (58.9%)	0.533
	501- 1000 JD	145 (44.5%)	181 (55.5%)	
	> 1000 JD	85 (40.3%)	126 (59.7%)	
education	Primary	38 (39.6%)	58 (60.4%)	0.155
	Secondary	3 (23.1%)	10 (76.9%)	
	High school	89 (42.6%)	120 (57.4%)	
	Under graduate	240 (44.7%)	297 (55.3%)	
	Post graduate	62 (35.6%)	112 (64.4%)	
Work	Part time job	58 (42.3%)	79 (57.7%)	0.118
	Full time	208 (40.55%)	306 (59.5%)	
	House wife	124 (44.9%)	152 (55.1%)	
	Don’t work	15 (62.5%)	9 (37.5%)	
	Retired	27 (34.6%0	51 (65.4%)	
Previous treatment	None	23 (30.7%)	52 (69.3%)	0.175
	Surgery	216 (42.9%)	287 (57.1%)	
	Radiotherapy	2 (40%)	3 (60%)	
	Hormonal therapy	39 (50%)	39 (50%)	
	Chemotherapy	152 (41.5%)	214 (58.5%)	
	Immunotherapy	0 (0)	2 (100%)	
Current treatment	None	76 (34.1%)	147 (65.9%)	0.122
	Surgery	128 (43.5%)	166 (56.5%)	
	BMT	3 (37.5%)	5 (62.5%)	
	Radiotherapy	65 (40.1%)	97 (59.9%)	
	Hormonal therapy	18 (51.4%)	17 (48.6%)	
	Chemotherapy	118 (46.8%)	134 (53.2%)	
	Immunotherapy	24 (43.6%)	31 (56.4%)	
Cancer Stage	Early	221 (40.8%)	321 (59.2%)	0.361
	Late	199 (43.6%)	257 (56.4%)	
Treatment status	Not under current treatment	94 (36.4%)	164 (63.6%)	0.041
	Under current treatment	338 (43.8%)	433 (56.2%)	
Have you previously received the influenza vaccine?	Yes	85 (41.5%)	120 (58.5%)	0.875
	No	347 (42.1%)	477 (57.9%)	
Do you take the influenza vaccine yearly?	Yes	129 (44.2%)	163 (55.8%)	0.369
	No	303 (41.15%)	434 (58.9%)	
Are you aware of the different COVID-19 vaccines?	Yes	151 (27.9%)	390 (72.1%)	<0.001
	No	281 (57.6%)	207 (42.4%)	
Do you know about the side effects of the vaccine?	Yes	97 (38.5%)	155 (61.5%)	0.212
	No	335 (43.1%)	442 (56.9%)	
Have you been previously infected with the COVID-19 Virus?	Yes	65 (51.2%)	62 (48.8%)	0.025
	No	367 (40.7%)	535 (59.3%)	
Are you worried about getting the virus in the future?	Yes	284 (36.8%)	488 (63.2%)	<0.001
	No	83 (63.8%)	47 (36.2%)	
Do you think that the vaccine will stop you from acquiring the virus	Yes	167 (32.2%)	352 (67.8%)	<0.001
	No	265 (52%)	245 (48%)	
Do you think that the success of the vaccine will positively affect your life?	Yes	187 (32%)	397 (68%)	<0.001
	No	245 (55.1%)	200 (44.9%)	
Which of the following reasons will drive you to take COVID-19 vaccine?	Fear of getting infected or death	187 (66.8%)	93 (33.2%)	<0.001
	Enhance Education and awareness	95 (45.7%)	113 (54.3%)	
	Peer encouragement	56 (17.6%)	263 (82.4%)	
If you are not willing to take the vaccine, Do you think your decision to take the vaccine may be affected after proper education?	Yes	151		NA
	No	281		

As for vaccine hesitancy, a different pattern emerged when analyzing the data. Participants who expressed a willingness (intent) to take the vaccine exhibited a higher level of knowledge and awareness of the different types of vaccines, as compared to those who reported being hesitant (72.1% vs. 27.9%, p<0.001).

The former, as compared to the latter group, also had a lower likelihood of previous COVID-19 infection or diagnosis (48.8% vs. 51.2%, p=0.025), higher levels of fear of COVID-19 infection (63.2% vs. 36.8%, p<0.001), and greater trust in the vaccine’s ability to protect them from the infection (67.8% vs. 32.2%, p<0.001).

Perception of future risk was another factor that influenced vaccine hesitancy and acceptance. Patient who expressed worry about contracting the virus in the future were more likely to accept the vaccine. Beliefs about the vaccines’ efficacy at preventing virus acquisition and its positive impact on people’s lives also influenced acceptance. Interestingly, proper education on the vaccine seemed to be associated with stronger vaccine acceptance. For example, many patients who initially expressed hesitancy about the vaccine were willing to take it after receiving proper education. Finally, other factors such as fear of infection or death represented primary motivators of vaccine acceptance in the study. This was followed by the desire to enhance education and awareness, and peer encouragement affected vaccine acceptance as well.

Overall, these data highlight the complex interplay of factors influencing COVID-19 vaccine acceptance, including knowledge and awareness, fear, beliefs, personal experiences, and social influences. Understanding these factors can help inform strategies to address vaccine hesitancy and promote vaccination acceptance.

Although results reported in **Table 3** reveal that willingness (intent) to take the COVID-19 vaccine by different diagnosis group were not statistically significant (P-value 0.069), the breakdowns by these diagnosis groups suggests that vaccine hesitancy varied notably across certain groups. For instance, the patients with Pancreatic Cancer demonstrated the highest hesitancy rate at 62.5%, followed by Breast Cancer (46.9%), GI Cancer (45.1%), and Head & Neck Tumors (48.1%). Conversely, patients with Gynecological cancers had the lowest hesitancy rate at 28.6%, while the others fell within the range of 34.0% to 50.0%, including Lung Cancer (34.3%), Eye Tumors (36.8%), Leukemia (37.5%), Brain & CNS Tumors (34.0%), Urology (40.2%), and Orthopedics & Spine (50.0%). This variation points to a potential need to better tailor diagnosis-specific approaches to these groups, in order to reduce vaccine hesitancy and improve acceptance.

**Table 3:** COVID-19 vaccine acceptance by cancer diagnosis group among patients of King Hussein Cancer Center, Jordan (Feb-April 2021).

Variables			Won’t take COVID 19 Vaccine (432)	Will take COVID 19 vaccine (597)	TOTAL	*P*
Diagnosis Grouped	Breast Cancer	Count (%)	122 (46.9%)	138 (53.1%)	260	0.069
	Urology	Count (%)	66 (40.2%)	98 (59.8%)	164	
	GI Cancer	Count (%)	69 (45.1%)	84 (54.9%)	153	
	Brain & CNS Tumor	Count (%)	32 (34.0%)	62 (66.0%)	94	
	Gynecology	Count (%)	24 (28.6%)	60 (71.4%)	84	
	Orthopedics & Spine	Count (%)	37 (50.0%)	37 (50.0%)	74	
	Lung cancer	Count (%)	24 (34.3%)	46 (65.7%)	70	
	Head & Neck Tumors	Count (%)	26 (48.1%)	28 (51.9%)	54	
	Leukemia	Count (%)	12 (37.5%)	20 (62.5%)	32	
	Eye Tumor	Count (%)	7.0 (36.8%)	12 (63.2%)	19	
	Pancreatic Cancer	Count (%)	5 (62.5%)	3 (37.5%)	8	
	Others	Count (%)	8 (47.1%)	9 (52.9%)	17	

In the multivariable regression model (**Table 4**) significant predictors of vaccine acceptance included: positive life changes, peer encouragement, fear of death or getting infected with COVID-19, enhancement of education and awareness, not under current treatment, and awareness of the different COVID-19 vaccine options. For example, positive life changes were found to be associated with a higher likelihood of willingness (intent) to take the vaccine (Odd ratio (OR) = 1.828, p < 0.001), as was peer encouragement; it also played a significant role in increasing vaccine acceptance among cancer patients (Wald Chi-square= 82.202, p < 0.001). Similar observations were made for enhancing education and awareness, and fear of death or getting infected (OR = 0.352, OR = 0.121, respectively) (p < 0.001).

**Table 4:** Multivariable (Binary Logistic) regression model of modifiable factors associated with COVID-19 hesitancy, King Hussein Cancer Center, Jordan (Feb-April 2021).

Outcome: Vaccine acceptance	Regression Coefficient	WALD	P-value	Odds Ratio (95% CI)
Positive life changes	.603	12.656	<0.001	1.828 (1.311-2.548)
Peer Encouragement		82.202	<0.001	–
Fear of death or get infected with COVID-19	-2.116	82.120	<0.001	0.121 (0.076-0.191)
Enhancement of Education & awareness	-1.043	22.514	<0.001	0.352 (0.229-0.542)
Not under Current treatment	.979	23.708	<0.001	2.662 (1.795-3.947)
Awareness of the different COVID-19 vaccines	.722	16.520	<0.001	2.059 (1.453-2.917)
Constant	.374	2.798	.094	1.453 (-)

(Vaccine acceptance were adjusted for the significant factors in the univariate analysis as follow: treatment status, awareness of different types of vaccine, positive life changes, peer encouragement, fear of death or being infected with COVID-19, & enhancement of Education & awareness).

## 4 Discussion

The prevention of infections is crucial for patients with impaired immunity, such as those with cancer, as infections can lead to higher morbidity and mortality rates (1). Despite the apparent benefits of immunization in preventing infections, many cancer patients are hesitant to receive vaccines. Currently, there is a lack of published data on COVID-19 vaccine hesitancy or acceptance specifically among cancer patients in Jordan. This study aimed to identify various factors that contribute to vaccine hesitancy in this particular population, some of which overlap with factors reported in general population surveys while others are unique to cancer patients.

This study sheds light on the willingness (intent) of cancer patients in Jordan to take the COVID-19 vaccine. It highlights the need to consider both disease-specific factors and modifiable factors when addressing vaccine hesitancy and acceptance in this vulnerable population. Understanding the drivers behind vaccine intention can help inform strategies to increase acceptance rates among cancer patients, ensuring their protection against COVID-19 and reducing associated risks.

The findings of this study reveal that over half of the surveyed cancer patients (n = 597; 58%) expressed willingness (intent) to receive the COVID-19 vaccine. Their acceptance rates are similar to those reported among cancer patients in Lebanon and Tunisia, where acceptance rates were 55% and 50.5% respectively (10, 11). The study identified both non-modifiable disease-specific factors and modifiable factors that influence the decision-making process. Interestingly, there was low heterogeneity observed across different demographic groups, indicating that demographic factors may not significantly impact vaccine intention, contrary to what has been reported in the literature (11, 12). The decision to receive the vaccine seemed to be driven more by necessity, considering factors such as pandemic severity, vaccine safety and efficacy data, and government policies.

Study findings indicated that patients with early-stage disease showed higher willingness (intent) to take the COVID-19 vaccine, as compared to those with late-stage disease (59.2% vs. 56.4%), but this difference, however, was not statistically significant (p=0.361).

These findings are similar to a study conducted in Hong Kong, which also failed to definitively demonstrate significant differences in vaccine acceptance among their participants at different stages of cancer (13). Nonetheless, a systematic review conducted by Prabani et al, 2022 (14), found that patients with advanced stages of cancer (stages III and IV) had lower acceptance of the COVID-19 vaccine. Another study conducted on cancer patients in Turkey showed that patients with stage IV cancer had significantly higher levels of vaccination fear compared to patients with stage II cancer (15).

These mixed findings in the literature may be attributed to cultural differences and awareness gaps among various study participants. Cultural differences are known to influence attitudes and beliefs towards vaccination, and variation in level of awareness can affect perceptions of vaccine benefits and risks.

Another important finding of the present study was that the decision to get vaccinated among cancer patients was largely influenced by treatment status. A majority of patients who were not under treatment were willing (intent) to take the vaccine more so than those who were undergoing treatment; this differs from findings by Brko et al., 2021, which indicated that 75% of cancer patients in Serbia who were in the active cancer treatment phase, early or metastatic stage did not receive the vaccine (16). Research by Heudel et al., 2021, found that less than 10% percent of cancer patients undergoing active treatment refused to get vaccinated (17). Again, variations in these findings suggest hidden roles of cultural differences in determining vaccine acceptance, some of which reflect the uncertainty about vaccine efficacy and safety throughout the COVID19 pandemic for those patients in ongoing active treatment. Clearly, patients who had more knowledge about the vaccine options were more likely to get the vaccine, highlighting the importance of proper education and awareness for these cancer patients. Suggesting that empowering physicians to provide the critical brief advice could be lifesaving. The healthcare sector could implement priority programming to help facilitate access to the COVID-19 vaccines to high-risk cancer patients, supporting physicians to more routinely provide information about COVID-19 and encouraging vaccination (18). The importance of having healthcare professionals promote vaccination and reduce vaccine hesitancy has been highlighted in the emerging evidence base, including the previously reported findings of Villarreal-Garza et al., 2021 (19).

The study findings exhibit that there was a minimal impact of proper health education on the decision-making process with a slight self-predicted increase in agreeability among patients (25%) upon combating misinformation. Another study on the impact of education on cancer patients showed increased agreeability with the vaccine and a heightened belief in efficacy, safety, and advocacy (20). A Polish survey reported that education and marital status were both significantly associated with willingness (intent) to take the COVID-19 vaccine (14); although these factors were not associated with similar patient willingness (intent) in our study. Geographic and cultural differences may have played a role in these inconsistent findings.

Our study found that a significant percentage of participants (67.8% and 68%) who were willing to receive the COVID-19 vaccine believe in the vaccine’s efficacy and anticipated success (p < 0.001). This finding aligns with those of Brodziak et al., which showed that a positive attitude towards getting vaccinated was critical for acceptance among the majority of Polish patients enrolled in their study (73.7%) (21). A considerable body of literature emphasizes the importance of building proper knowledge and understanding through official campaigns and credible spokespersons (22). In the our study, 45.6% of participants expressed the value of peer encouragement on influencing their decision-making. This finding is consistent with the research of Jarrett et al. they showed and highlighted the role of the social system in increasing education and awareness (22). These findings and results also underscore the potentially vital role that social media and community engagement can play in diminishing vaccine hesitancy and increasing acceptance. Media and social media campaigns are known as potent tools for disseminating information and educating the public, especially vaccine information that can be trusted and is accurate. Trusted community sources and support groups are other tools that can further foster trust among cancer patients, and thereby help debunk vaccine-related misinformation. A 2020 study by Wilson & Wiysonge found a strong correlation between organizing activities on social media and public skepticism towards vaccine safety. The study documented a significant relationship between foreign disinformation campaigns and a decline in vaccination coverage (3).

The pandemic’s psychosocial impact on cancer patients is another factor to consider when thinking about ways to improve vaccine uptake. During the health crisis, it was evident that most cancer patients exhibited a higher level of generalized anxiety and specific concerns about death. During the pandemic, fear and anxiety played significant roles in influencing patients’ willingness (intent) to get vaccinated. A substantial percentage (63.2%) of our study participants expressed fear towards being infected with COVID-19, a factor that likely drove many of our cancer patients’ decisions about the COVID-19 vaccine. By understanding the powerful role fear plays in shaping vaccine acceptance, the medical and public health communities can develop and better tailor, more inclusive public health campaigns and interventions to address vaccine hesitancy and improve acceptance among cancer patients. These findings align well with previous research by Erdem et al., 2022, where they demonstrate that a majority (86.7%) of cancer patients who accepted the vaccine had heightened anxiety towards the virus, as measured by the COVID-19 phobia scale (C19P-S) (15).

The present study also points to the importance of peer-led education programs in reaching and helping unvaccinated patients to get vaccinated. This program approach may be underused in vaccine campaigns and could help address some of the observed vaccine hesitancy reasons in cancer patients. Our study also found that previous COVID-19 infection was associated with a decreased likelihood of vaccine acceptance. This association could be attributed to the presumption of long-term immunity after recovery and reduced fear among those who had been previously infected.

Future research and COVID-19 vaccine campaigns should consider these various factors identified in our study. Among the key research needs might be the need to conduct follow-up studies so that changes in attitudes and vaccine acceptance among cancer patients could be documented as these individuals recover from the pandemic. Capturing this information could provide valuable insights into the evolving dynamics of cancer patients’ needs and strategies that health systems are developing or using to address vaccine hesitancy and acceptance in this vulnerable population.

### 4.1 Strengths and limitations

The present survey study possesses several notable strengths that contribute to its robustness. Firstly, the large sample size employed in the research facilitates a fairly comprehensive representation of cancer patients with diverse malignancies. As such, study findings could be generalizable to a broader range of cancer patients. In addition, the inclusion of a heterogeneous mix of different cancer disease types ALSO enhanced our study’s ability to capture the nuances and variations in vaccine acceptance across the various cancer diagnoses. Furthermore, the utilization of a multistage data analysis approach added depth and rigor to the methodology used, allowing for a more thorough exploration of the different factors that are influencing vaccine acceptance among the cancer patients in Jordan. These strengths collectively enhance the reliability and applicability of the study’s findings. However, it is important to acknowledge several limitations of the study. First, the data collection occurred during a specific phase of the COVID-19 pandemic, and the study may not fully capture the evolving dynamics of the health crisis. The introduction of new vaccines and the dissemination of updated information may have led to shifts in cancer patients’ perspectives on vaccination, potentially rendering our findings less generalizable to later stages of the pandemic. Furthermore, the study focused on a specific geographical region and may not encompass the diversity of perspectives and experiences of cancer patients in different contexts. Additionally, the data were self-reported, which introduces the possibility of recall bias and social desirability bias. While we made efforts to mitigate these biases, they remain inherent limitations of survey-based research. Finally, the study is cross-sectional, which limits our ability to establish causal relationships or capture the potential changes in attitudes over time.

## 5 Conclusions

Efforts to combat the COVID-19 pandemic and facilitate recovery have accelerated the development and usage of effective COVID-19 vaccines. However, vaccine hesitancy, potentially resulting in low acceptance rates, continues to pose a risk, prolonging the severity and impact of COVID-19 on patients with active malignancies. In our study, we identified awareness about vaccines, fear of infection, and peer encouragement as pivotal modifiable factors associated with increased vaccine acceptance (reduced hesitancy) among cancer patients at our medical center in Jordan. Recognizing and understanding these modifiable factors provide oncologists and healthcare providers with pathways to address vaccine hesitancy by offering personalized advice, resources, and healthcare interventions to cancer patients. Moreover, it allows providers to establish better trust with their patients regarding vaccine safety, side effects, and appropriate usage. These factors are globally relevant and can be integrated into government (e.g., public health) guidelines to optimize COVID-19 vaccination uptake at national and regional levels. They can also guide the development of more effective peer-led educational campaigns aimed at enhancing confidence and trust in vaccines, especially among patients with active malignancies—the most vulnerable group concerning this respiratory infection.

## Data availability statement

The original contributions presented in the study are included in the article/supplementary material. Further inquiries can be directed to the corresponding author.

## Ethics statement

The studies involving humans were approved by Institutional Review Board/King Hussein Cancer Center (IRB-KHCC). The studies were conducted in accordance with the local legislation and institutional requirements. The participants provided their written informed consent to participate in this study.

## Author contributions

RA: Conceptualization, Methodology, Writing – original draft, Writing – review & editing. MM: Conceptualization, Methodology, Writing – original draft, Writing – review & editing. RD: Data curation, Formal Analysis, Writing – original draft. KA: Formal Analysis, Writing – review & editing. YS: Formal Analysis, Writing – original draft, Writing – review & editing.
